# The distributions of protein coding genes within chromatin domains in relation to human disease

**DOI:** 10.1186/s13072-019-0317-2

**Published:** 2019-12-05

**Authors:** Enrique M. Muro, Jonas Ibn-Salem, Miguel A. Andrade-Navarro

**Affiliations:** Institute of Organismic and Molecular Evolution, Johannes Gutenberg University of Mainz, Hans-Dieter-Hüsch-Weg 15, 55128 Mainz, Germany

**Keywords:** Chromatin structure, Topologically associating domains, TAD, Human diseases, Genes associated with disease, Housekeeping genes, Chromatin interactions, Enhancers, Gene regulation

## Abstract

**Background:**

Our understanding of the nuclear chromatin structure has increased hugely during the last years mainly as a consequence of the advances in chromatin conformation capture methods like Hi-C. The unprecedented resolution of genome-wide interaction maps shows functional consequences that extend the initial thought of an efficient DNA packaging mechanism: gene regulation, DNA repair, chromosomal translocations and evolutionary rearrangements seem to be only the peak of the iceberg. One key concept emerging from this research is the topologically associating domains (TADs) whose functional role in gene regulation and their association with disease is not fully untangled.

**Results:**

We report that the lower the number of protein coding genes inside TADs, the higher the tendency of those genes to be associated with disease (*p*-value = 4 × $10^{-54}$). Moreover, housekeeping genes are less associated with disease than other genes. Accordingly, they are depleted in TADs containing less than three protein coding genes (*p*-value = 3.9 × $10^{-34}$). We observed that TADs with higher ratios of enhancers versus genes contained higher numbers of disease-associated genes. We interpret these results as an indication that sharing enhancers among genes reduces their involvement in disease. Larger TADs would have more chances to accommodate many genes and select for enhancer sharing along evolution.

**Conclusions:**

Genes associated with human disease do not distribute randomly over the TADs. Our observations suggest general rules that confer functional stability to TADs, adding more evidence to the role of TADs as regulatory units.

## Background

The chromatin structure in Eukarya exceeds, in causes and consequences, just to be the product of packing long DNA polymers into a tiny nucleus volume; i.e., 2 m of DNA into a $5\,\upmu \text{m}$ diameter nucleus in human. Chromatin structure is a key factor in many biological functions like transcription, chromosome translocations, DNA repair and replication [1]. Lessening the spatial distance between two loci that are far in the linear genome can trigger a functional after-effect. Enhancer–promoter interactions are a paradigm of this and even inter-chromosomal interactions have been observed [2]. Enhancers are not distributed in a gene-centric way, with their genome locations correlating rather with TADs [3]. In accordance, 49% of the enhancers are in a range of 120 Kbp to the target promoter, from those only a 15% regulate the closest gene and only 56% regulate at least one of the closest five genes [4]. A classic example of a genomic distal interaction is the activation of shh by means of the ZRS enhancer, which is located within an intron of another gene,  1 Mbp away in mouse. ZRS is preferred over closer enhancers [5, 6]. Artificial modifications of the chromatin structure have been observed to have functional consequences, like, for example, the activation of the already inactive $\upbeta$-globin without its canonical transcription factor GATA1 in erythroblasts [7].

Different techniques have been used to unravel the hierarchical architecture of the chromatin [8, 9]. Chromosome conformation capture (3C) detects close genomic regions (three dimensional space) by proximity ligation using formaldehyde-mediated cross-linking [10]. Those loci-captures are quantified by means of a sequencing technology, determining the technology variant (4C, 5C, Hi-C and ChIA-PET). The recent Hi-C [11–13] stands out, incorporating next-generation high-throughput sequencing and calculating the capture frequency in an all-to-all manner, that is, for any pair of genomic loci. The Hi-C data provided from Rao et al. on Human GM12878 cells [13] reached a resolution of less than 1 Kbp, at the cost of 4.9 billions reads for a single experiment.

One of the main conclusions derived from 3C-based techniques is that the genome is partitioned in regions that have high levels of self-interaction contacts and can be distinguished from flanking genomic regions, the so-called contact domains or TADs. In human, they have a size that ranges from 40 Kbp to 3 Mbp, with a median value of 185 Kbp [13]. TADs are well conserved between different cell types and mammal species, especially between syntenic regions [1, 12–15], clearly indicating their functional role.

TADs have, surrounding their borders, insulators that limit the action of enhancers outside the TAD. Modifying the shh enhancer–promoter linear genomic distance within the TAD by means of genome engineering does not modify shh expression. On the contrary, without a strong TAD context, shh expression depends on the linear genomic distance to its enhancer [16].

There is no such structured chromatin at early stages of development; the structural order arises during embryogenesis in the same time-frame as gene expression starts in the zygote. The main features of the structure will be maintained after a certain structural stability is reached. How chromatin structure conformation arises is unknown but emerges from TAD borders triggered by architectural proteins and factors like Zelda in *Drosophila melanogaster* [17] or CTCF and cohesin in mammals [18].

On the other hand, housekeeping genes (HKs) are constitutively expressed in all tissues and are necessary for basal cellular function. With respect to the structure of the chromatin, HK transcription starting sites (TSSs) have a strong preference to be located at TAD boundaries [12].

An additional angle onto the study of the functional role of TADs regards the pathologic effects of the disruption of their structure. It has been long known that disrupting the regulatory loop that conforms the enhancer–promoter interactions might be pathogenic [19, 20], also affecting brain development [21, 22]. More specifically, structural variations affecting TAD borders can cause an ectopic reorganization leading to gene aberrant misexpression, and therefore to disease [23, 24]. Some disruptions are well studied, like the one affecting the regulation of Pax3 by means of CRISPR/Cas9, which gives rise to a limb bud malformation [23]. But, the relationship between TAD structures and human diseases is not fully elucidated. To shed light on this topic, we decided to study the distribution of disease related genes within TADs. Our observations show that the disease-associated genes do not distribute randomly across TAD structures.

## Results

### The distance to the TAD border

**Fig. 1:**
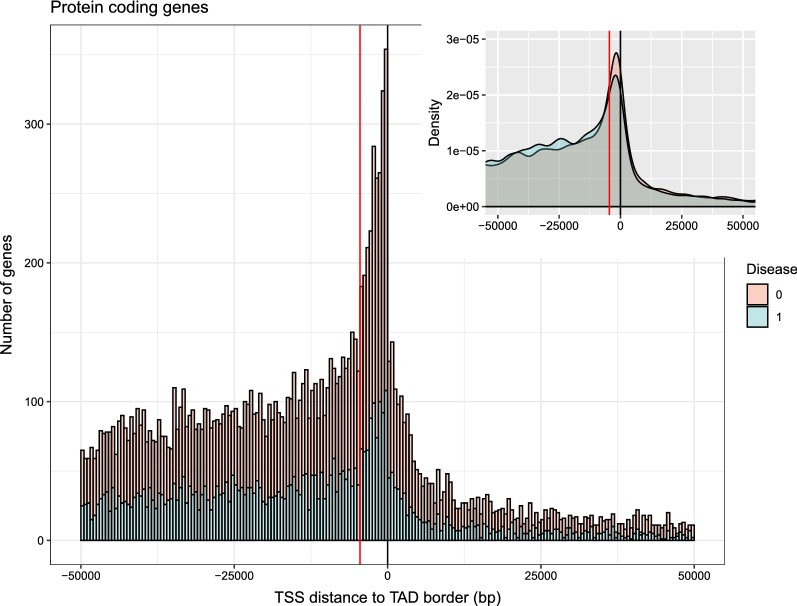
Distributions of the distances from the TSSs of the genes to their corresponding closest TAD borders. If the TSS is not within a TAD, the distance is calculated to the closest TAD border. Negative and positive distances correspond to TSSs inside and outside TADs, respectively. The vertical black line represents the TAD borders. Two different distributions are shown in the main plot where genes associated and non-associated with disease are represented in blue and salmon color, respectively. An abrupt enrichment of TSSs is observed within TADs starting at a distance of − 4500 nt within TADs (indicated with a vertical red line). The portion of the genome within TADs is 55% (see "Methods"). Each bin of the histogram represents 500 nt. Inset: the density for the same data is shown. Even if TSSs have a preference toward the TAD borders, the preference is lower for genes associated with disease (*p*-value = 7.37 × $10^{-10}$, Wilcoxon rank test)

We analyzed the distribution of distances from the TSSs of the genes to their closest TAD borders depending on their association with disease (human GM12878; see "Methods"). TSSs near TAD borders are less associated with disease (Fig. 1; *p*-value = $7.37\times10^{-10}$, Wilcoxon rank test). Thanks to the high resolution of the Hi-C data set, we were able to detect an abrupt enrichment of TSSs within TADs from the borders up to a distance of − 4500 nt (Fig. 1; [12]), as well as the already known HKs bias toward TAD borders (Additional file 1: Figure S1;* p*-value = 3 × $10^{-4}$, Wilcoxon rank test). The fact that genes near TAD borders are less associated with disease is in agreement with the preference of loci of HK for TAD borders: HK genes (genome wide) are less associated with disease than non-HK genes (30.9% and 36.6%, respectively; Table 1, *p*-value = $1.67\times10^{-10}$). A plausible explanation is that since HKs are expressed in all tissues and are relevant for cell survival, alterations of these genes would be less tolerated by the organism than those of the rest of genes, thus the fewer HKs associated with disease. Independently of that, the tendency observed in Fig. 1 can also be seen in both HK and non-HK genes, which are less associated with disease at TAD borders (Additional file 2: Figure S2).

**Table 1:** HK genes are less associated with disease than non-HK genes, a *p*-value = $1.67\times10^{-10}$ from a Chi-square test has been obtained

	Disease	Non-disease
HK	1129	2521
Non-HK	5305	9186

### Dependence between the number of genes associated with disease and the number of genes contained in TADs

**Fig. 2:**
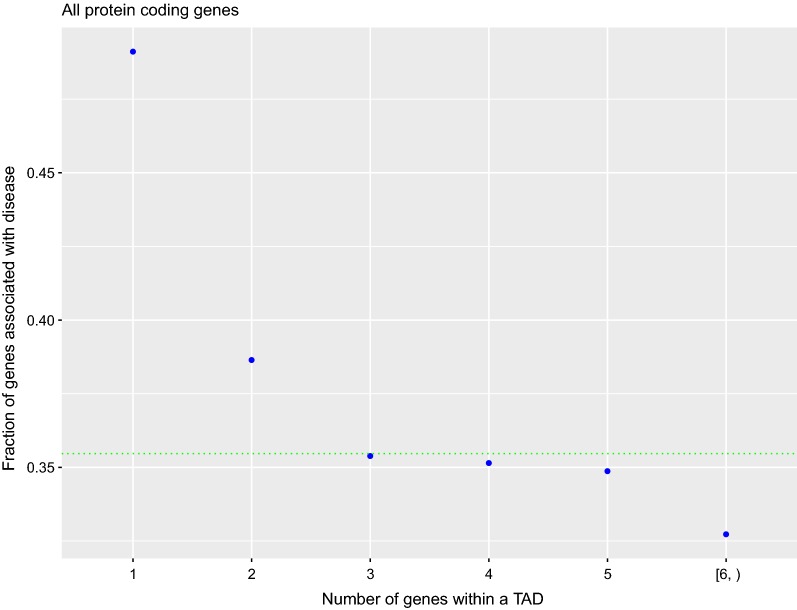
Fraction of genes associated with disease (ordinates) depending on the number of genes contained within the TADs (abscissas); the numbers have been aggregated for $n \ge 6$. The lower the number of genes inside the TAD the higher fraction of the genes associated with disease. A *p*-value = 4 × $10^{-54}$ from a Chi-square test, comparing the number of genes associated and non-associated with disease for the six TAD categories, has been obtained. The green dotted line represents the genome-wide fraction of genes associated with disease (0.354)

Next, we categorized the TADs by the number of genes they contain in six different categories (see "Methods"). From all the 9274 TADs, 2934 TADs (31.6%) have a unique gene within them, 1717 (18.5%) have two genes, 878 TADs (9.4%) have three, 552 TADs (5.9%) have four, 343 ( 3.6%) have five, and 833 TADs (8.9%) have six or more genes within them (Additional file 3: Figure S3). We observed inverse variation between the fraction of genes associated with disease and the size of the TAD categorized by the number of genes it contains (Fig. 2; *p*-value = $4\times10^{-54}$, Chi-square test). While the genome-wide fraction of genes associated with disease is 0.35 (see the green dotted line), this fraction is significantly larger in TADs with one or two genes. In particular, for the 2934 TADs containing only one gene it is 0.49 (Additional file 4: Table S4).

It must be noticed that the fraction of genes associated with disease is 5.1% lower when comparing the set of 4718 genes that are within no TAD with the 13,423 genes contained within TADs; the fractions are 31.7% and 36.8% for genes outside and within TADs, respectively. We analyzed if the difference could be due to an annotation bias on genes outside TADs. For that we counted how many genes lack any Gene Ontology annotation [25] in both sets. The comparison shows significant results (*p*-value = $7.9\times10^{-4}$, Chi-square test): genes outside TADs have a higher percentage of genes with no GO annotation (5.6% in comparision with the 4.4% of genes within TADs), but the difference between both sets is small and can not explain the lower fraction of genes associated with disease we observed for genes outside TADs. To carry out further analysis on genes outside TADs, we tried to observe if they follow a trend like the one shown in Fig. 2, associating each gene with its closest TAD, but no trend was observed.

**Fig. 3:**
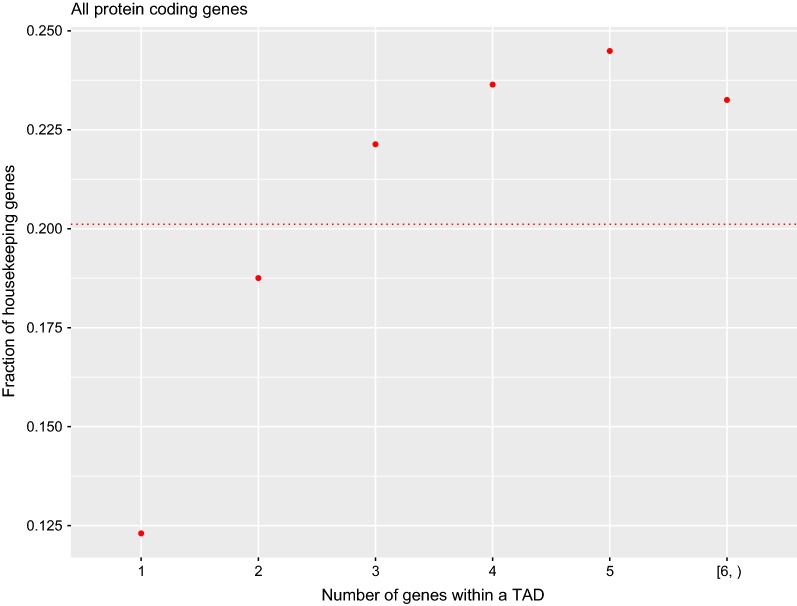
Fraction of HKs, that is HKs/(HKs + non-HKs), categorized by the number of genes within their TADs. HKs are not homogeneously distributed over the different TAD categories. The red dashed line indicates the genome-wide fraction of HKs (0.201)

Next, in accordance with the enrichment of disease genes in TADs containing fewer genes and the lower association of HKs with disease (Fig. 2 and Table 1, respectively), HKs were significantly depleted in TAD categories containing fewer genes (Fig. 3; *p*-value = $1.2\times10^{-39}$, Chi-square test). We have also proved that this distribution of HKs over TADs is not the driving force of the inverse variation between the fraction of genes associated with disease and the number of genes the TADs contain (Fig. 2), because the trend is observed independently both for HK and non-HK genes (see Additional file 5: Figure S4).

We hypothesized that the observed inverse variation between the fraction of genes associated with disease and the number of genes within the TAD (Fig. 2) is related to the TAD regulatory complexity. To further investigate this, we obtained the 49,672 enhancers identified in the GM12878 cell line from EnhancerAtlas [26], see "Methods". Although it is difficult to know with confidence which enhancer targets which gene promoter, most of the enhancers that regulate a gene are within the same TAD that contains the regulated gene [16, 27]. We observe that while TADs with more genes also contain more enhancers (Additional file 6: Figure S5), the number of enhancers per gene decreases in those TADs (Additional file 7: Figure S6).

**Fig. 4:**
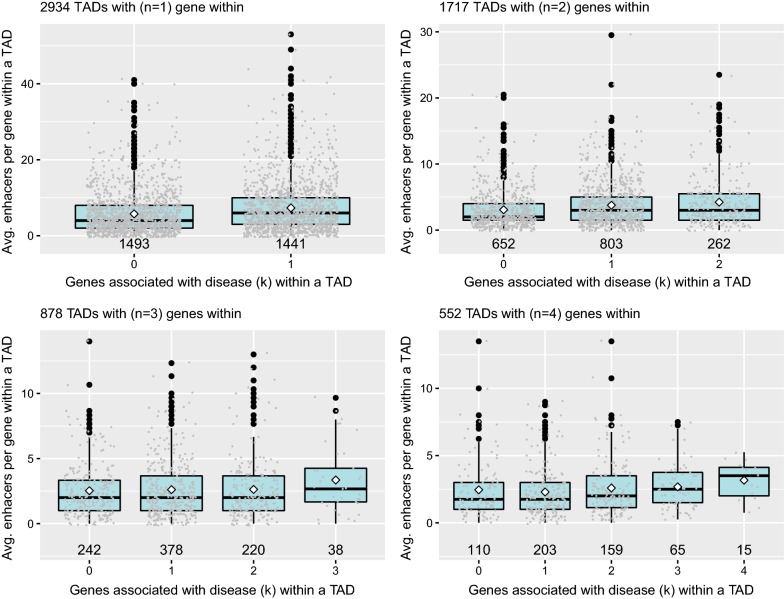
For different TAD categories (with *n* = 1, 2, 3 or 4 genes within), the distributions of the average numbers of enhancers per gene within the TADs are represented depending on the number of genes associated with disease within the TAD (*k*; where $0 \le k \le n$). White diamonds and horizontal black lines show the median and mean of the boxplots, respectively. Values of $n \ge 5$ were not considered due to the low number of occurrences

We studied this variation in more detail. We observed for each TAD category, depending on the number of genes they contain, that the average number of enhancers per gene increases in TADs with more genes associated with disease (Fig. 4; *p*-values are $3.2\times10^{-12}$, $4.8\times10^{-6}$, $6.5\times10^{-2}$ and $2.9\times10^{-2}$; Wilcoxon rank test). In addition, TADs with fewer genes have a higher ratio of enhancers to genes (Additional file 8: Figure S7).

Collectively, our results show that, when examining TAD composition, there seems to be a correlation between higher average number of enhancers per gene and higher fraction of disease genes within the TADs. In addition, we observed that TADs with fewer genes have a higher fraction of genes associated with disease (Fig. 2). Although it is complicated to establish cause and effect relationships between these observations, for each category of TADs, the average number of enhancers per gene is also increasing with the fraction of genes associated with disease. We conclude that TADs with many genes tend to share regulatory enhancers and that results in a lower number of disease genes.

## Discussion

Research that focused on the three-dimensional structure of the chromatin is suggesting biological function that goes beyond the mere packaging of the polymer into the nucleus. It is known that TAD reorganization, following TAD border disruptions can lead to disease [23, 24]. But, the relation between the chromatin structure and disease is not yet fully elucidated. In this work, we studied the distribution of protein coding genes within TADs to find functional properties of TADs in relation to human disease not previously described in the literature. We wondered if there would be further hints supporting the function of TADs at a level more intimately related to gene function. It is admitted that besides the conservation of tandem duplicated genes [28], clusters of obviously related genes, such us the Hox genes, are more the exception than the rule.

We observed that the TSS of genes that locate towards the TAD borders have a lower tendency to be associated with disease (Fig. 1). The known preference of gene TSSs for TAD borders with a stronger bias for HKs ( [12]; Fig. 1 and Additional file 1: Figure S1) led us to analyze the association of HKs with disease. As a result, we found that HKs are less associated with disease than non-HKs (Table 1; *p*-value = $1.67\times10^{-10}$). A plausible explanation is that since HKs are expressed in all tissues and are relevant for cell survival, mutations in HKs would be evolutionarily selected out. In accordance with this result, it is already known that HKs are more evolutionarily conserved than tissue specific [29, 30].

To find a relation between the fraction of genes associated with disease and a simple physical property of TADs, we chose the number of genes the TAD contains, which relates to gene regulation and allowed us to categorize TADs depending on a simple discrete argument.

We observed that TADs containing a lower amount of genes are more prone to carry genes associated with disease (Fig. 2). TADs containing a unique gene have the highest fraction of gene association with disease, a 13.6% more than the average genome-wide gene association with disease. This is an indication that TADs containing more genes are functionally more stable. In addition, we observed that while TAD length increases with the number of genes contained within TADs (Additional file 9: Figure S8), there is no relation between the fraction of genes associated with disease and the length of the TADs. We take these results as an indication that the number of TSSs within TADs captures information about gene regulation within TADs better than TAD length.

Interestingly, and due to the fact that HKs are less associated with disease, we also observed that HKs do not distribute uniformly all over the different TAD categories, and have a significant tendency to be located within TADs containing more than two gene TSSs (Fig. 3). This result is in agreement with the previously found genomic co-localization of HK clusters [31]. Nevertheless, HKs are not the driving force of the trend observed in Fig. 2, because the same trend has been found independently for both HK and non-HK genes (Additional file 5: Figure S4).

All these associations were observed in contact domains predicted by the Arrowhead algorithm for Hi-C data from B-lymphoblastoid cell line [13]. From the many computational methods developed to detect TADs, the Arrowhead is one of the most respected, specially for the detection of hierarchical TAD structures with a high resolution; see the following references for comparisons of algorithms [32–34]. Nevertheless, we would like to point out that the Arrowhead obtains relatively small TADs in comparison with other algorithms. Moreover, in our analysis, we showed that it covers a 55% of the whole genome. Also, a 26% of the total amount of protein coding genes are within no TAD of this cell line, due to the fact that a gene is within a TAD only if its TSS is contained within it (see "Methods"). Then, for generalizing our results, it will be necessary to reproduce them using TADs predicted by means of different algorithms, different cell lines and development conditions. Moreover, even if the set of protein coding genes is the best studied in relation to human disease, it would be interesting to extend our work to different types of genes, as well as some other annotations that associate genes with human disease.

We also observed that TADs containing more genes have a higher total number of enhancers within the TADs, but a lower a ratio of enhancers versus number of genes within the TAD. We propose that the inverse variation observed in Fig. 2 is a consequence of gene regulatory complexity within TADs: TADs containing more genes have a tendency to share enhancers, ultimately resulting in a decreasing association of those genes with disease.

## Conclusion

Protein coding genes associated with human disease do not distribute randomly over the TADs. A significant higher association with disease is observed in TADs containing less than three genes. Housekeeping genes are less associated with disease and tend to be located within TADs containing at least two more genes. Our observations have implications in the understanding of human disease in relation to the distribution of human genes over the chromatin structure, adding evidence in support of TADs as regulatory units.

## Methods

### Protein coding genes

From all the different types of genes, this study focuses on protein coding genes because it is the type of gene that is best annotated in relation to human disease. A reliable and well-annotated set of protein coding genes has been used; from the set of 20,171 reviewed SwissProt human entries [35], we selected those 18,823 proteins with a high UniProt annotation score. That is, 14,871 and 3952 with evidence at the protein and transcript level, respectively. Those inferred from homology (649), predicted (121) or with uncertain evidence (578) were not considered.

A total of 18,395 protein coding Gene IDs, from the CTD comparative toxicogenomics database [36] are already mapped by UniProt to their corresponding SwissProt entries. CTD Gene IDs not annotated at the current NCBI gene annotation database [37] or that could not be mapped to Ensembl [38] or to Gencode v25 [39] were not considered (see below in this "Methods" section). Also, genes located at chromosome Y were discarded for consistency with the Hi-C data from a female donor cell line [13]. As a result, 18,141 reliable protein coding genes were considered (Additional file 10: Table S1). For each gene, one transcription start site (TSS) was considered, located at the 5′ end locus of the gene [27, 28].

### NCBI gene identifiers

A total of 20,703 human protein coding gene identifiers [37] were annotated and downloaded from the NCBI website. From those, 19,135 have a mapping to an ENSEMBL gene with ENSG-prefix.

### Mapping to ENSEMBL/gencode

We used Gencode v19 (July 2013; ENSEMBL 74, 75) as the latest release with annotations based on the GRCh37/hg19 genome version. For mapping genes, we used a more recent version, Gencode v25, with annotations on GRCh38/hg38 and the corresponding mappings to GRCh37/hg19 already provided. These mappings are based on the UCSC genome browser LiftOver [40] at UCSC file gencode.v25lift37.annotation.gtf.

### Genes associated with diseases

The comparative toxicogenomics database (CTD, update 2017 from http://ctdbase.org/ [36]) is nowadays a standard among the databases annotating genes associated with diseases. CTD manually curates annotations obtained from the peer-reviewed scientific literature and from the OMIM database [41]. The CTD database is well maintained and monthly updated since 2006. We filtered out gene-disease associations based on inferred data, considering only gene-disease associations with a direct evidence (6434 out of 18,141 genes analyzed (35.46%); Additional file 10: Table S1).

### Housekeeping genes

The detection of HKs is nowadays very accurate because of the RNA-seq technology. HKs were obtained from [42]. From our set of genes, 3650 (20.1%) are HKs (Additional file 11: Table S2).

### Topologically associating domains (TADs)

The human GM12878 contact matrix obtained for the B-lymphoblastoid cell line from the Hi-C experiments carried out by Rao et al. [13] is very accurate with 4.9 billion pairwise contacts at resolution of less than 1 Kbp. The Hi-C protocols and methods were based on previous developments by the same group [11]. For TAD detection, instead of the previous directionality index method [12], the Arrowhead algorithm was introduced [13]; it detects contact domains that are considered as TADs. The benefits of Hi-C experiments in terms of resolution and throughput, together with the new Arrowhead algorithm, led to the most precise TAD detection, with an unprecedented hierarchical organization and a reduction of the mean TAD size to 185 Kbp, from the previous 1 Mbp [12]. A comparison of different algorithms for TAD prediction can be found in [32–34].

As a result, 9274 GM12878 TADs are well annotated, on GRCh37/hg19, with a resolution lower than 1 Kbp (GEO accession id GSE63525).

TADs do not cover the whole genome. We have calculated the portion of the genome that is contained within those TADs. Chromosome annotations were obtained from the table chromInfo.txt (UCSC/hg19) and the chromosome Y was excluded from the analysis because the Hi-C data was obtained from a female donor. Moreover, the hierarchical organization of TADs was considered, not counting more than once in the case of TAD overlapping. As a result, 55% of the genome is contained within the 9274 TADs.

### The number of genes within TADs

Following previous studies [12, 28], we considered that a TAD contains a gene only if the TSS of the gene is within the TAD. As a result, from the total of 18,141 protein coding genes analyzed, 13,423 (74%) are within TADs of the B-lymphoblastoid cell line; that is, 4718 protein coding genes (26%) are within no TAD of this cell line. On the other hand, from the total of 9274 TADs obtained with the Arrowhead algorithm, 2,017 (21.7%) contain no TSS of any protein coding gene. But, most of those overlap another part of at least one gene (of any type); only 88 TADs ($<1$%) do not overlap any gene annotated by gencode v19.

### Distance from gene TSSs to their closest TAD borders

For each TSS contained within a TAD, and for each TAD containing any TSS, we calculated the distance (negative) from the TSS to the closest TAD boundary. For TSSs contained within no TAD, the distance (positive) to the closest TAD boundary is taken. 13,423 TSSs (74%) are within a TAD (Additional file 12: Table S3).

### Enhancers

We used the 49,672 enhancers specific to the GM12878 cell line and annotated on GRCh37/hg19 from EnhancerAtlas 2.0 [26].

## Supplementary information

**Additional file 1: Figure S1.** Distribution of the distances from the TSS of genes to their closest TAD borders. The TAD borders are represented with a vertical black line. Blue and salmon color represent HK and non-HK genes, respectively. If the TSS is within a TAD a negative distance is calculated, otherwise the distance is positive. Each bin represents 500 nt. Inset: the density for the same data is shown. The preference of HKs toward the TAD borders is significant (p-value = 3 × 10^−4^, Wilcoxon rank test).

**Additional file 2: Figure S2.** Distribution of the distances from the TSS of the genes to their closest TAD borders depending on the gene association with disease. The TAD border is represented with a vertical black line. Blue and salmon color represent genes associated and not with disease, respectively. If the TSS is within a TAD a negative distance is calculated, otherwise the distance is positive. a. HK genes. b. non-HK genes. Insets: The densities for the same data is shown. Genes not associated with disease have higher preference for TAD borders but this is only significant for non-HK genes (p-value = 9 × 10^−11^, Wilcoxon rank test).

**Additional file 3: Figure S3.** Number of TADs depending on the number of genes within the TADs. The counts are displayed behind each bar. Many TADs contain few genes and from a total of 9274 TADs, 2017 TADs (21.7%) have no gene within them.

**Additional file 4: Table S4.** TADs that contain only one gene.

**Additional file 5: Figure S4.** Fraction of genes for HK and non-HK genes associated with disease (ordinates) depending on the number of genes contained within the TADs (n; abscissas); the numbers have been aggregated for n ≥ 6. The lower the number of genes inside the TAD the higher fraction of the genes associated with disease: a. HK genes; a p-value = 3.6 × 10^−5^ from a Chi-square test, comparing the number of genes associated and non-associated with disease for the six TAD categories, was obtained. The green dotted line represents the genome-wide fraction of HK genes associated with disease (0.309). b. non-HK genes; a p-value = 1.2 × 10^−43^ from a Chi-square test has been obtained. The green dotted line represents the genome-wide fraction of non-HK genes associated with disease (0.366).

**Additional file 6: Figure S5.** Distribution of the number of enhancers within TADs versus the number of genes contained within the TADs. Mean and median values of each boxplot are shown by white diamonds and black horizontal lines, respectively. The more genes within a TAD, the larger the number of enhancers.

**Additional file 7: Figure S6.** Distribution of the ratios of the number of enhancers to genes depending on the number of genes within a TAD. Mean and median values of each boxplot are shown by white diamonds and black horizontal lines, respectively.

**Additional file 8: Figure S7.** Mean ratios of the number of enhancers per gene within the TADs versus the number of genes within the TAD associated with disease (0 ≤ k ≤ n), where n is the total number of genes within the TAD. The value of n, which determines the TAD category, is represented for TADs with n = 1, 2, 3, and 4 genes (red, blue, green and purple lines, respectively). TADs with fewer TSSs have higher ratios of enhancers to TSSs. Moreover, for each TAD category, the higher the number of genes associated with disease, the higher the average number of enhancers per gene.

**Additional file 9: Figure S8.** Distribution of TAD lengths depending on the number of TSSs they contain. An horizontal black line indicates the median for each TAD category.

**Additional file 10: Table S1.** The 18,141 different protein coding genes. Each row has the following information in the columns: geneid, gene locus, transcription starting site (TSS), and CTD gene association or not with disease.

**Additional file 11: Table S2.** The 3650 different protein coding HKs.

**Additional file 12: Table S3.** Distance of each TSS to the closest TAD border. The distance (negative) has been calculated for each TAD where the TSS is contained. If the TSS is within no TAD the closest distance (positive) to a TAD border has been calculated. Each entry of the table displays the following information by columns: geneId, gene strand, gene locus, TSS of gene, distance to the TAD border, and TAD.

## Data Availability

All the data used in this work are freely available in the corresponding Additional files.
