# Supplementary figures and images for: The distributions of protein coding genes within chromatin domains in relation to human disease
Source: Epigenetics Chromatin. 2019 Dec 5;12:72. doi: 10.1186/s13072-019-0317-2 (PMC6894242; doi:10.1186/s13072-019-0317-2)

# Protein coding genes

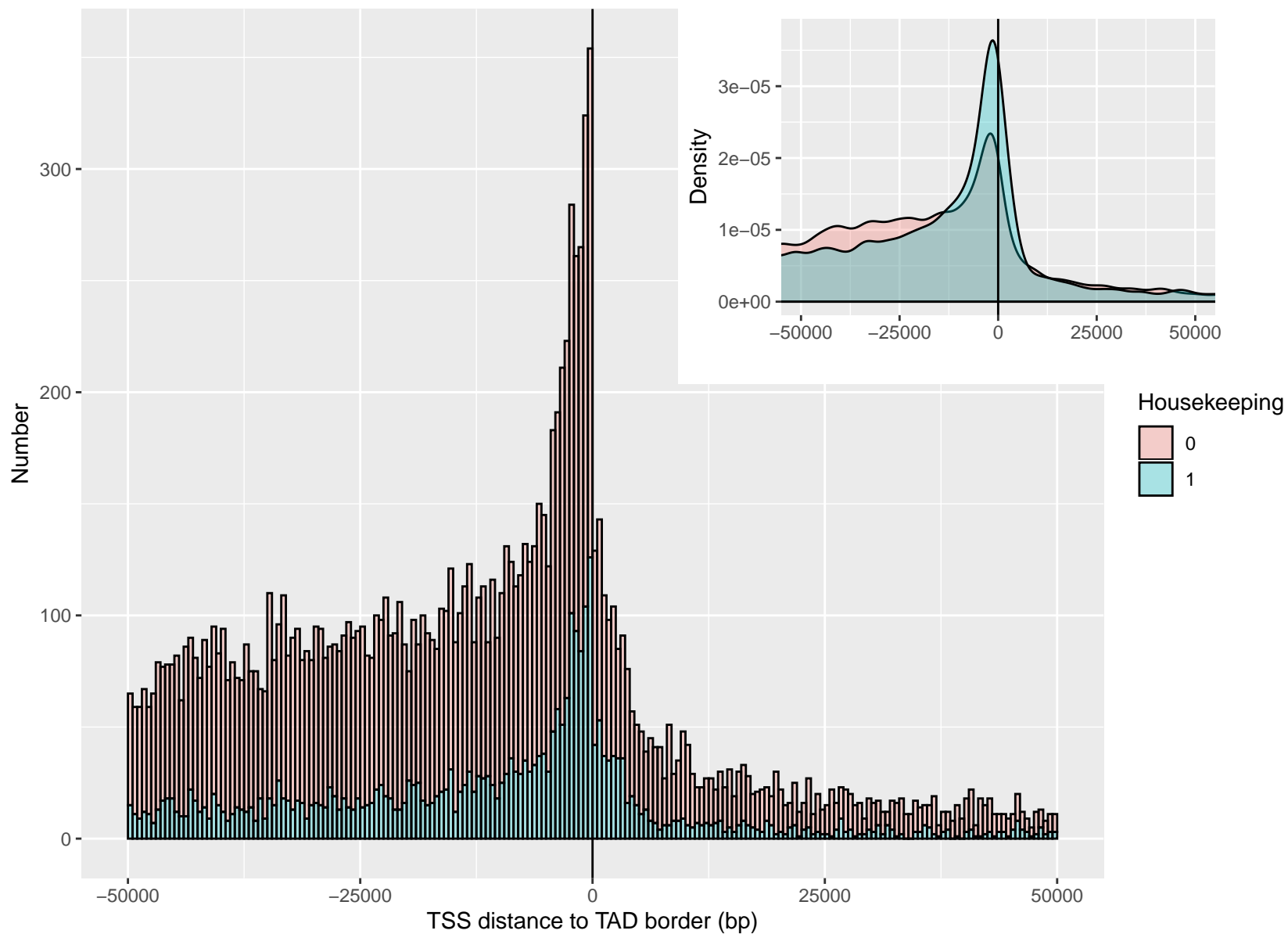

Supplement: Supplementary file 1 — Additional file 1: Figure S1. Distribution of the distances from the TSS of genes to their closest TAD borders. The TAD borders are represented with a vertical black line. Blue and salmon color represent HK and non-HK genes, respectively. If the TSS is within a TAD a negative distance is calculated, otherwise the distance is positive. Each bin represents 500 nt. Inset: the density for the same data is shown. The preference of HKs toward the TAD borders is significant (p-value = 3 × 10^−4^, Wilcoxon rank test). [file 13072_2019_317_MOESM1_ESM.pdf]

# Housekeeping genes

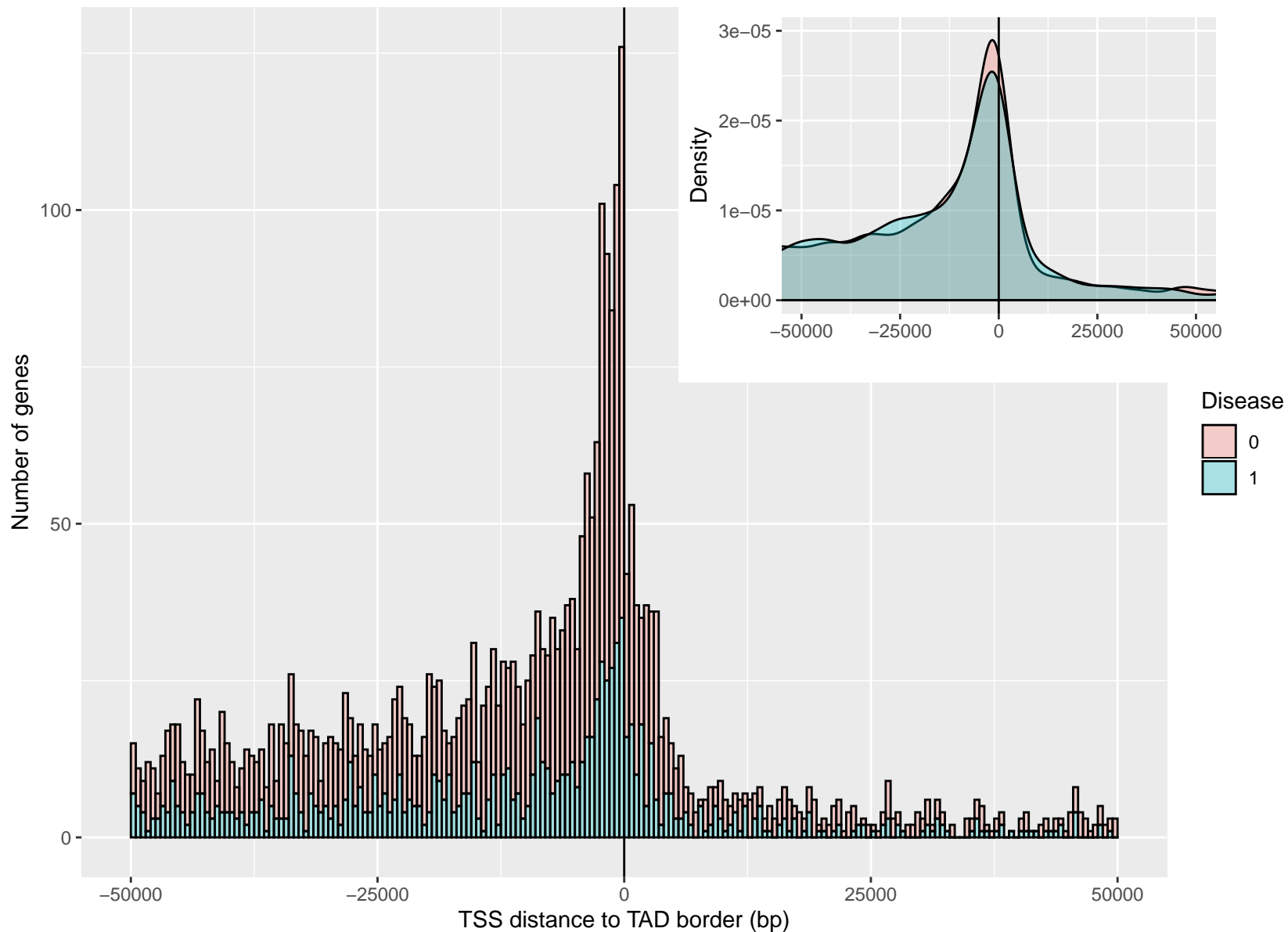

# Any protein coding gene but housekeeping

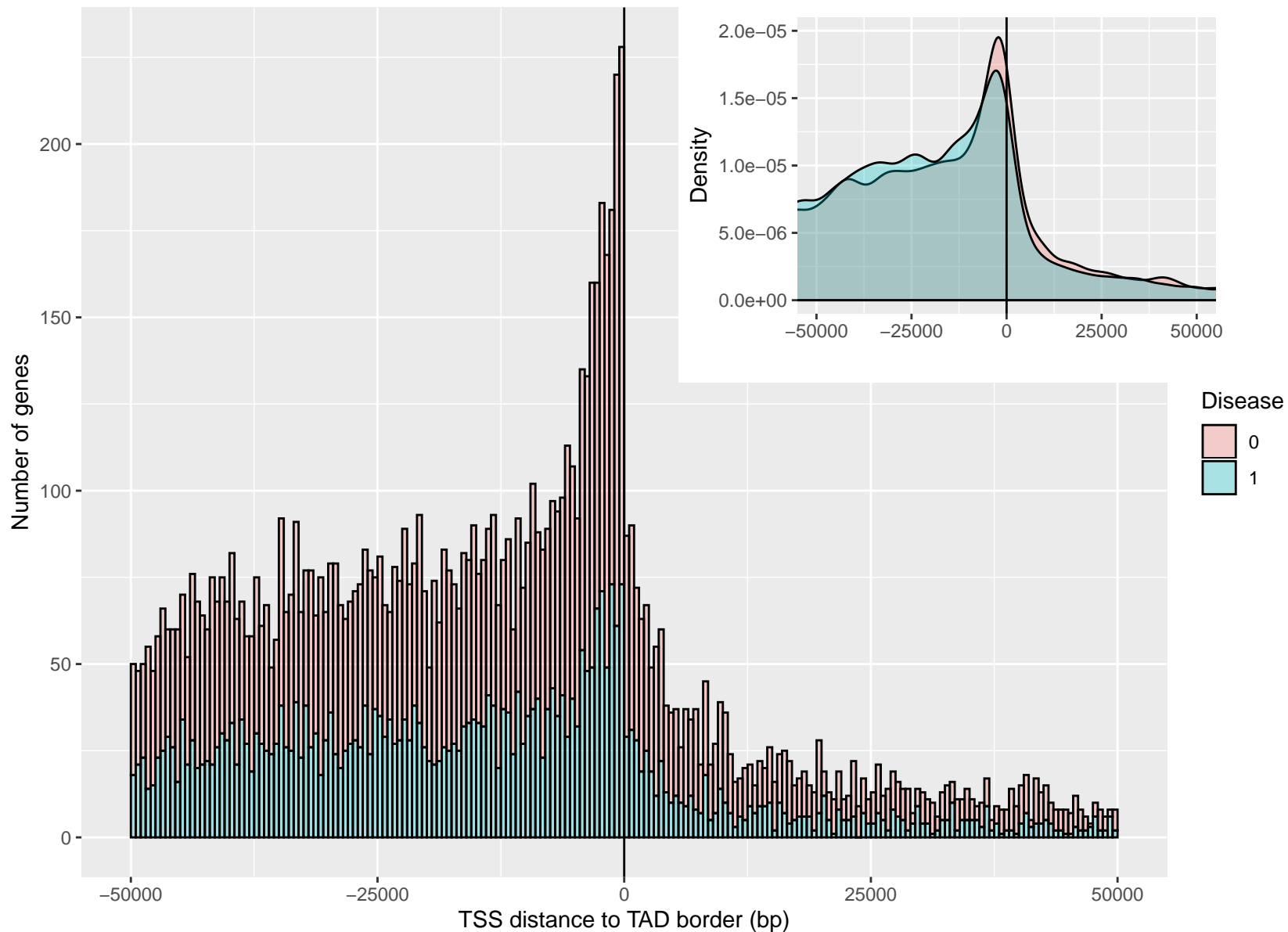

Supplement: Supplementary file 2 — Additional file 2: Figure S2. Distribution of the distances from the TSS of the genes to their closest TAD borders depending on the gene association with disease. The TAD border is represented with a vertical black line. Blue and salmon color represent genes associated and not with disease, respectively. If the TSS is within a TAD a negative distance is calculated, otherwise the distance is positive. a. HK genes. b. non-HK genes. Insets: The densities for the same data is shown. Genes not associated with disease have higher preference for TAD borders but this is only significant for non-HK genes (p-value = 9 × 10^−11^, Wilcoxon rank test). [file 13072_2019_317_MOESM2_ESM.pdf]

# All protein coding genes

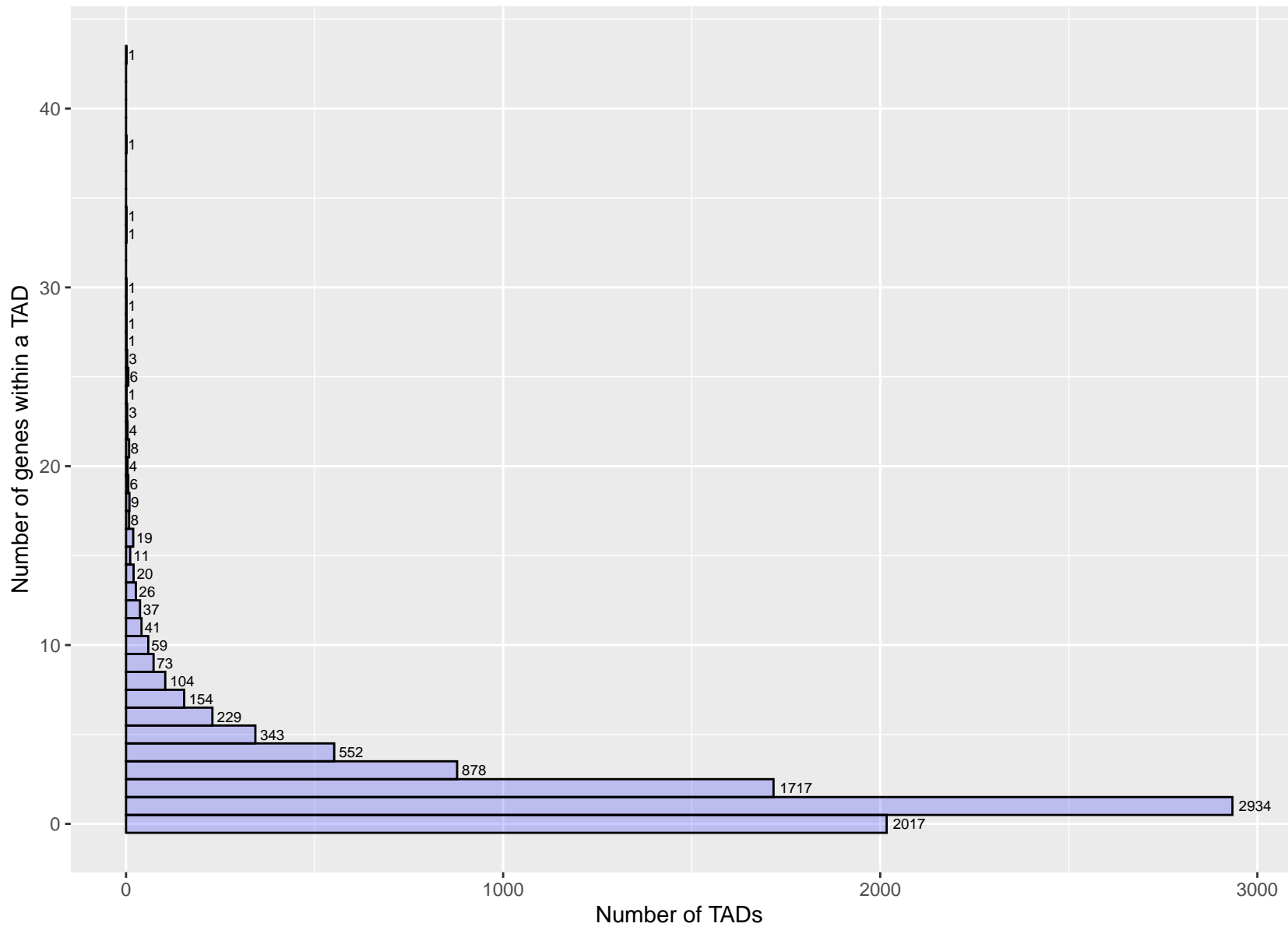

Supplement: Supplementary file 3 — Additional file 3: Figure S3. Number of TADs depending on the number of genes within the TADs. The counts are displayed behind each bar. Many TADs contain few genes and from a total of 9274 TADs, 2017 TADs (21.7%) have no gene within them. [file 13072_2019_317_MOESM3_ESM.pdf]

# Housekeeping genes

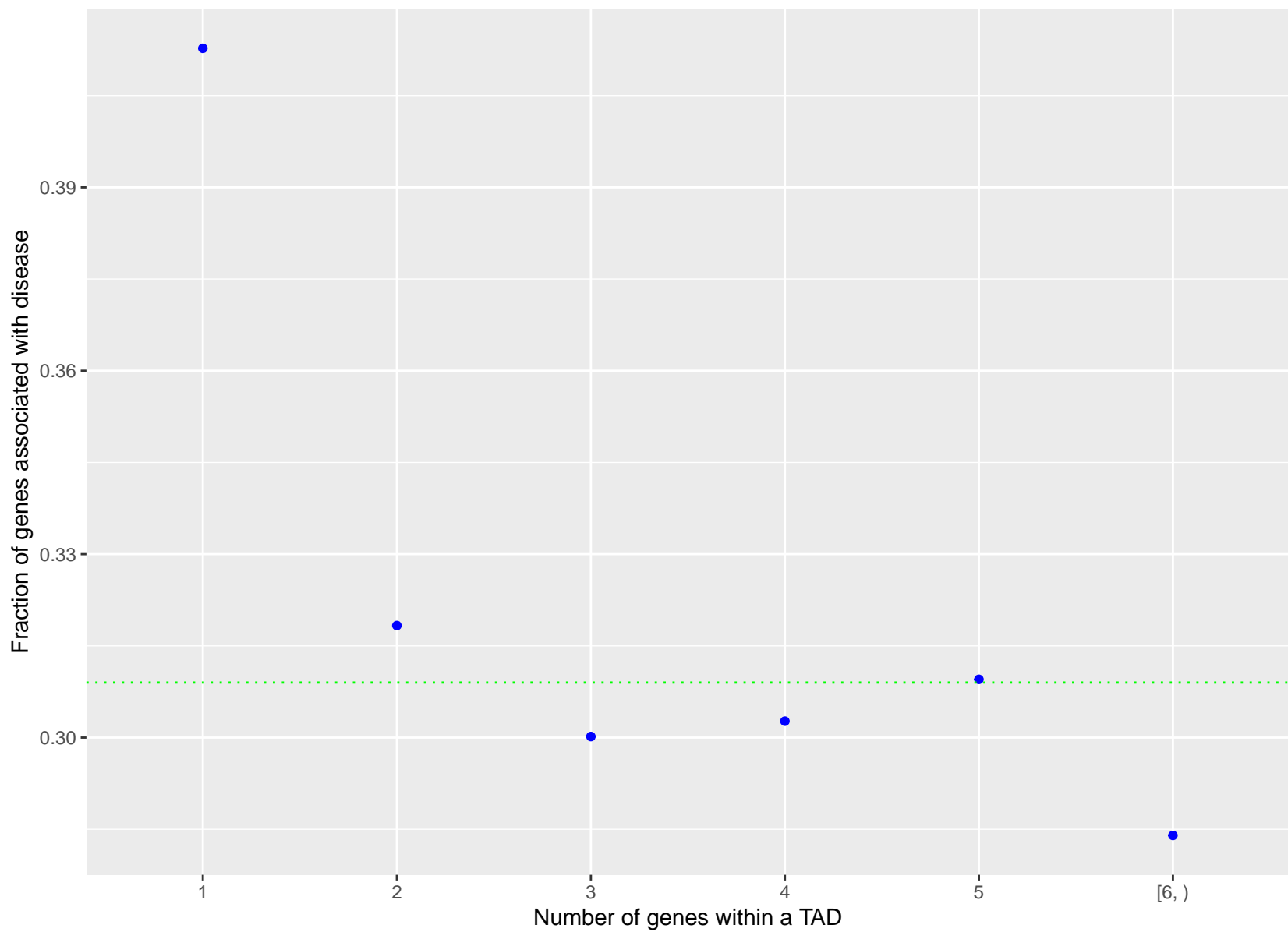

Any protein coding gene but housekeeping

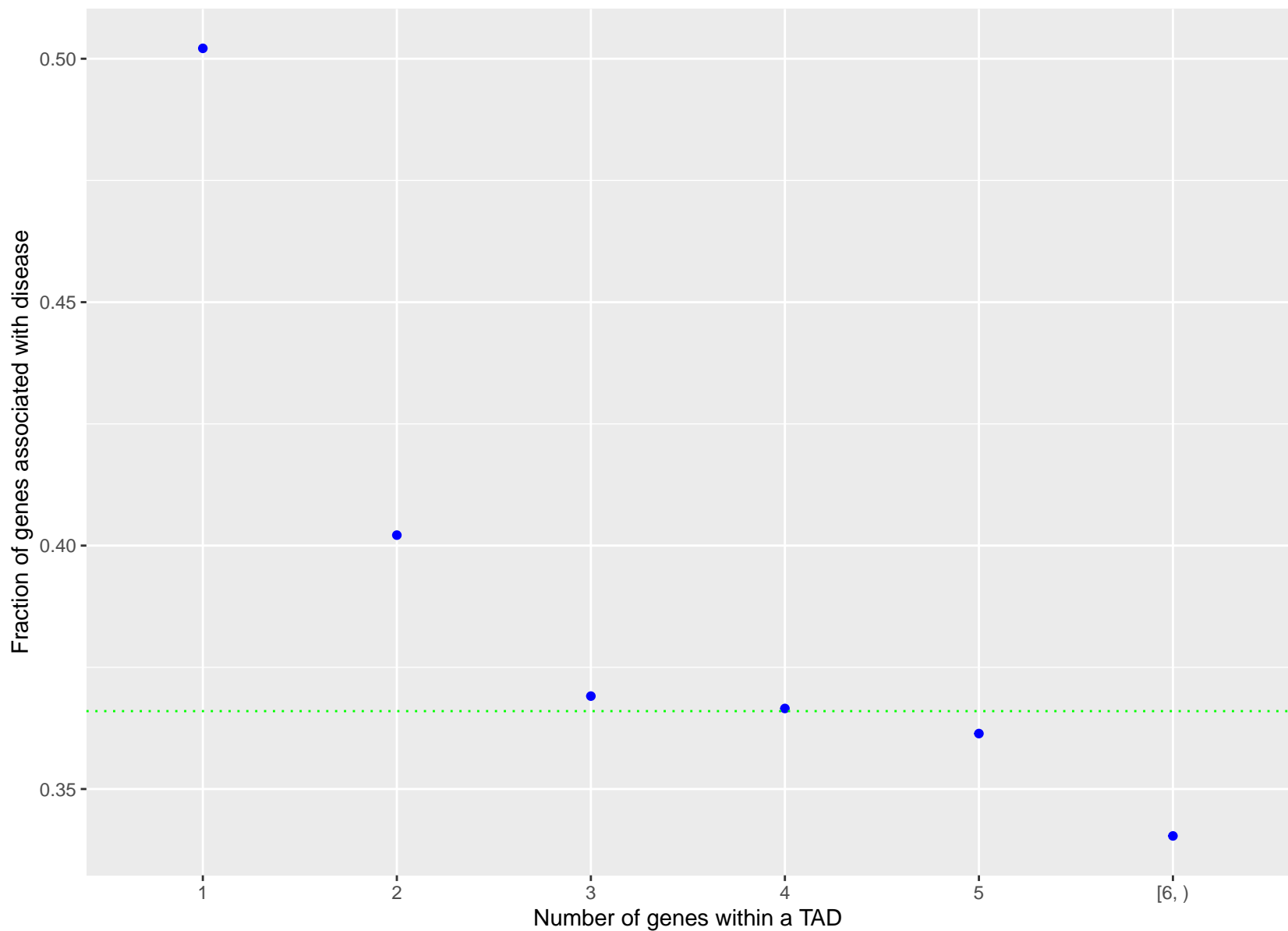

Supplement: Supplementary file 5 — Additional file 5: Figure S4. Fraction of genes for HK and non-HK genes associated with disease (ordinates) depending on the number of genes contained within the TADs (n; abscissas); the numbers have been aggregated for n ≥ 6. The lower the number of genes inside the TAD the higher fraction of the genes associated with disease: a. HK genes; a p-value = 3.6 × 10^−5^ from a Chi-square test, comparing the number of genes associated and non-associated with disease for the six TAD categories, was obtained. The green dotted line represents the genome-wide fraction of HK genes associated with disease (0.309). b. non-HK genes; a p-value = 1.2 × 10^−43^ from a Chi-square test has been obtained. The green dotted line represents the genome-wide fraction of non-HK genes associated with disease (0.366). [file 13072_2019_317_MOESM5_ESM.pdf]

# All protein coding genes

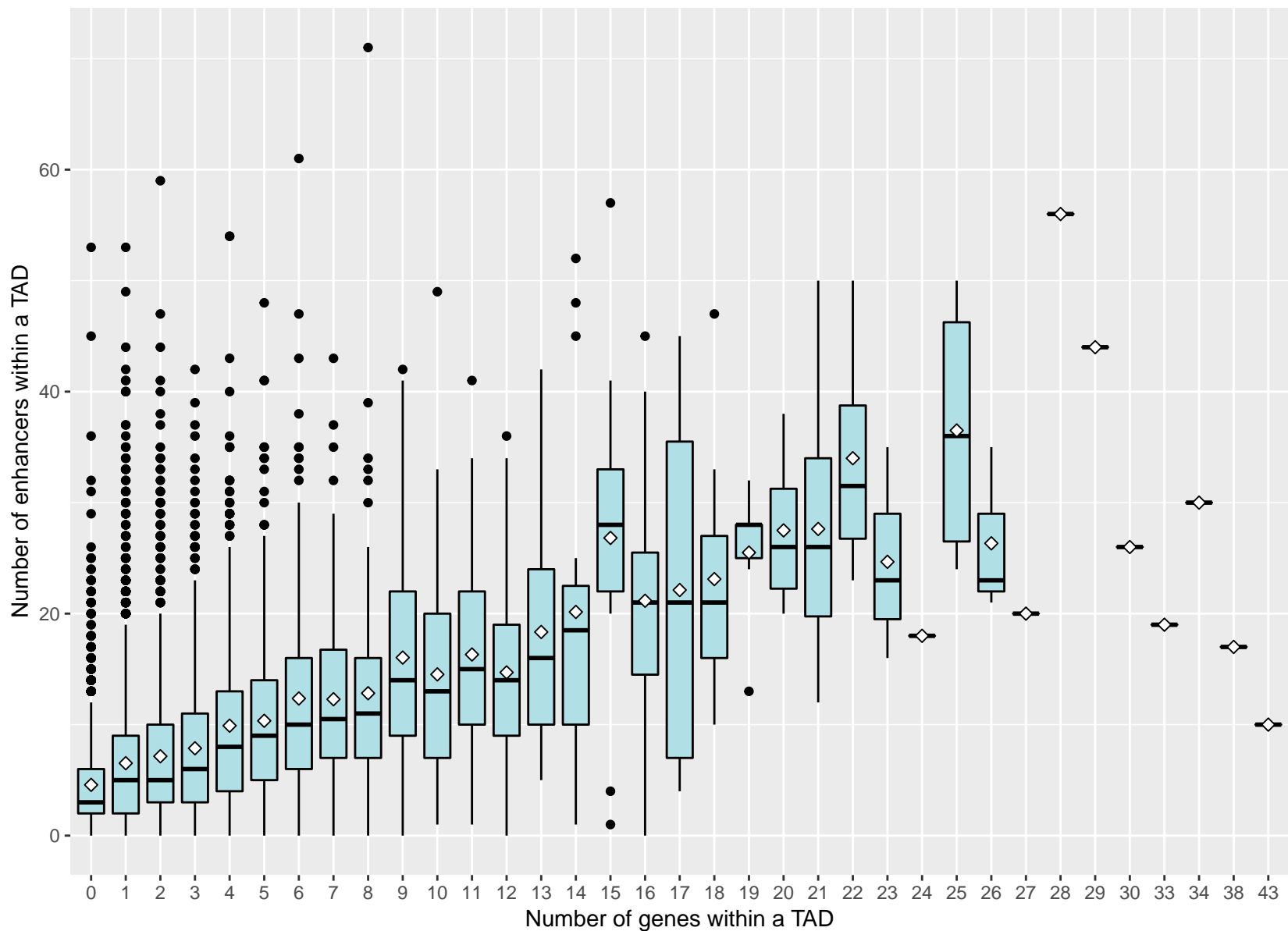

Supplement: Supplementary file 6 — Additional file 6: Figure S5. Distribution of the number of enhancers within TADs versus the number of genes contained within the TADs. Mean and median values of each boxplot are shown by white diamonds and black horizontal lines, respectively. The more genes within a TAD, the larger the number of enhancers. [file 13072_2019_317_MOESM6_ESM.pdf]

# All protein coding genes

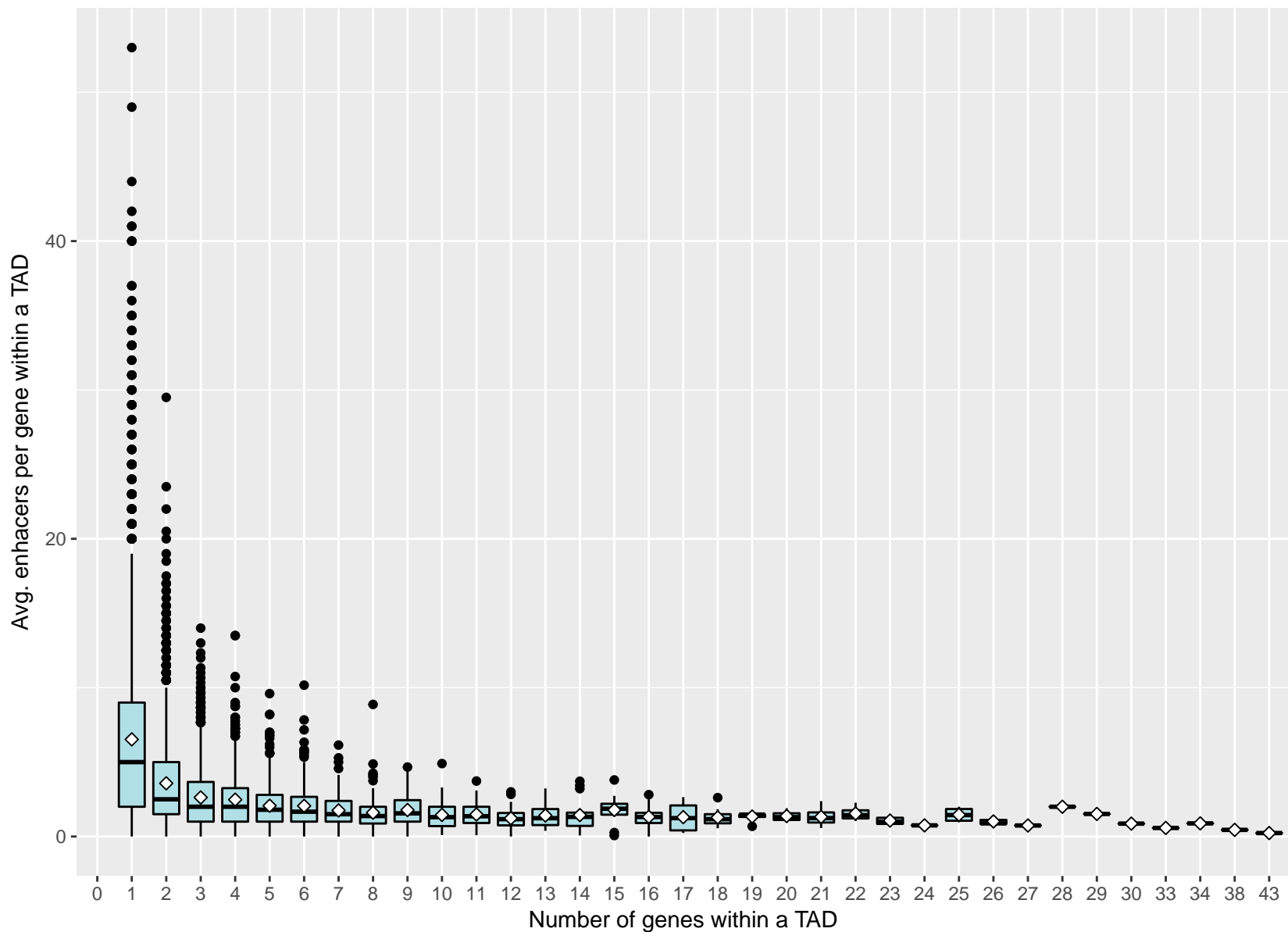

Supplement: Supplementary file 7 — Additional file 7: Figure S6. Distribution of the ratios of the number of enhancers to genes depending on the number of genes within a TAD. Mean and median values of each boxplot are shown by white diamonds and black horizontal lines, respectively. [file 13072_2019_317_MOESM7_ESM.pdf]

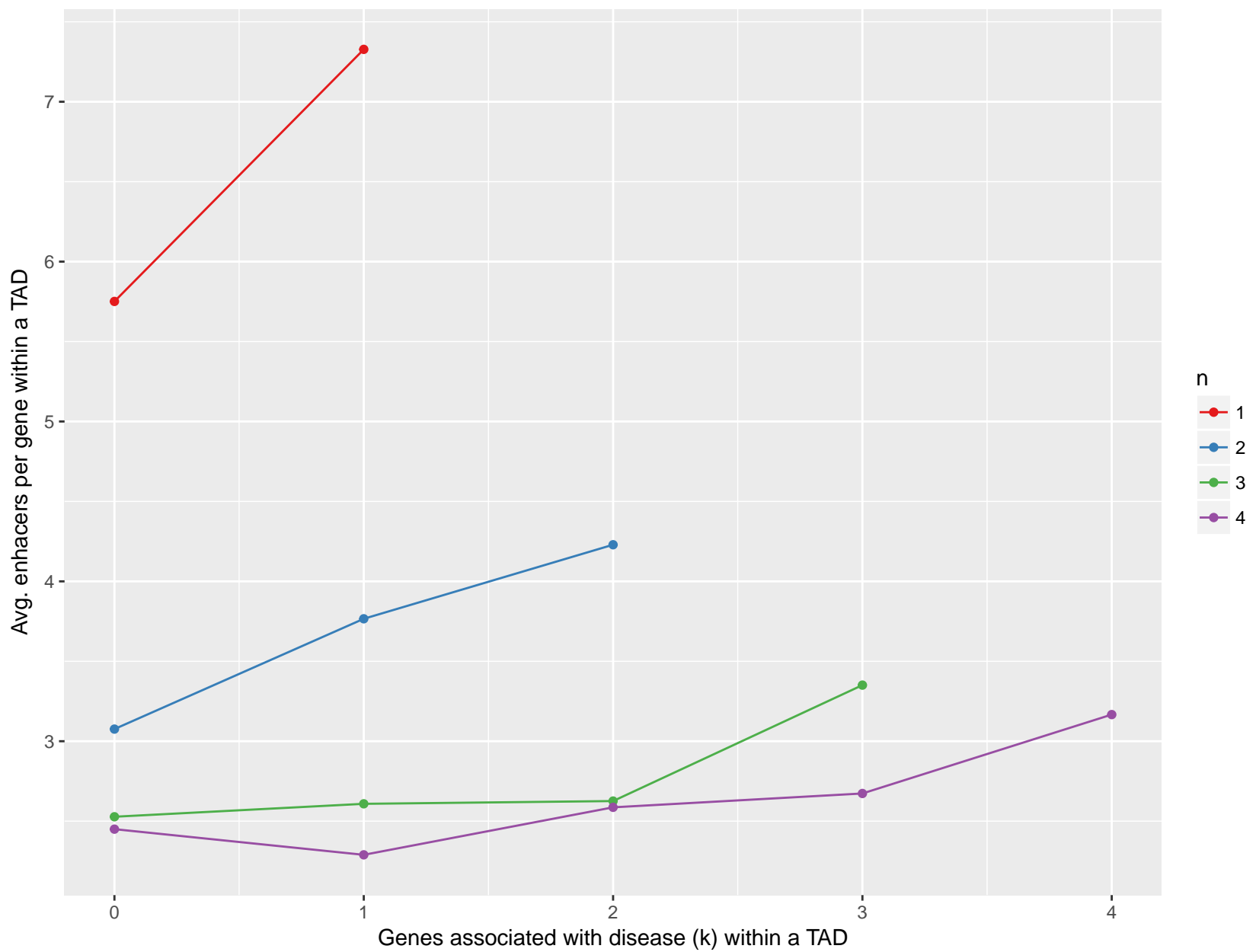

Supplement: Supplementary file 8 — Additional file 8: Figure S7. Mean ratios of the number of enhancers per gene within the TADs versus the number of genes within the TAD associated with disease (0 ≤ k ≤ n), where n is the total number of genes within the TAD. The value of n, which determines the TAD category, is represented for TADs with n = 1, 2, 3, and 4 genes (red, blue, green and purple lines, respectively). TADs with fewer TSSs have higher ratios of enhancers to TSSs. Moreover, for each TAD category, the higher the number of genes associated with disease, the higher the average number of enhancers per gene. [file 13072_2019_317_MOESM8_ESM.pdf]

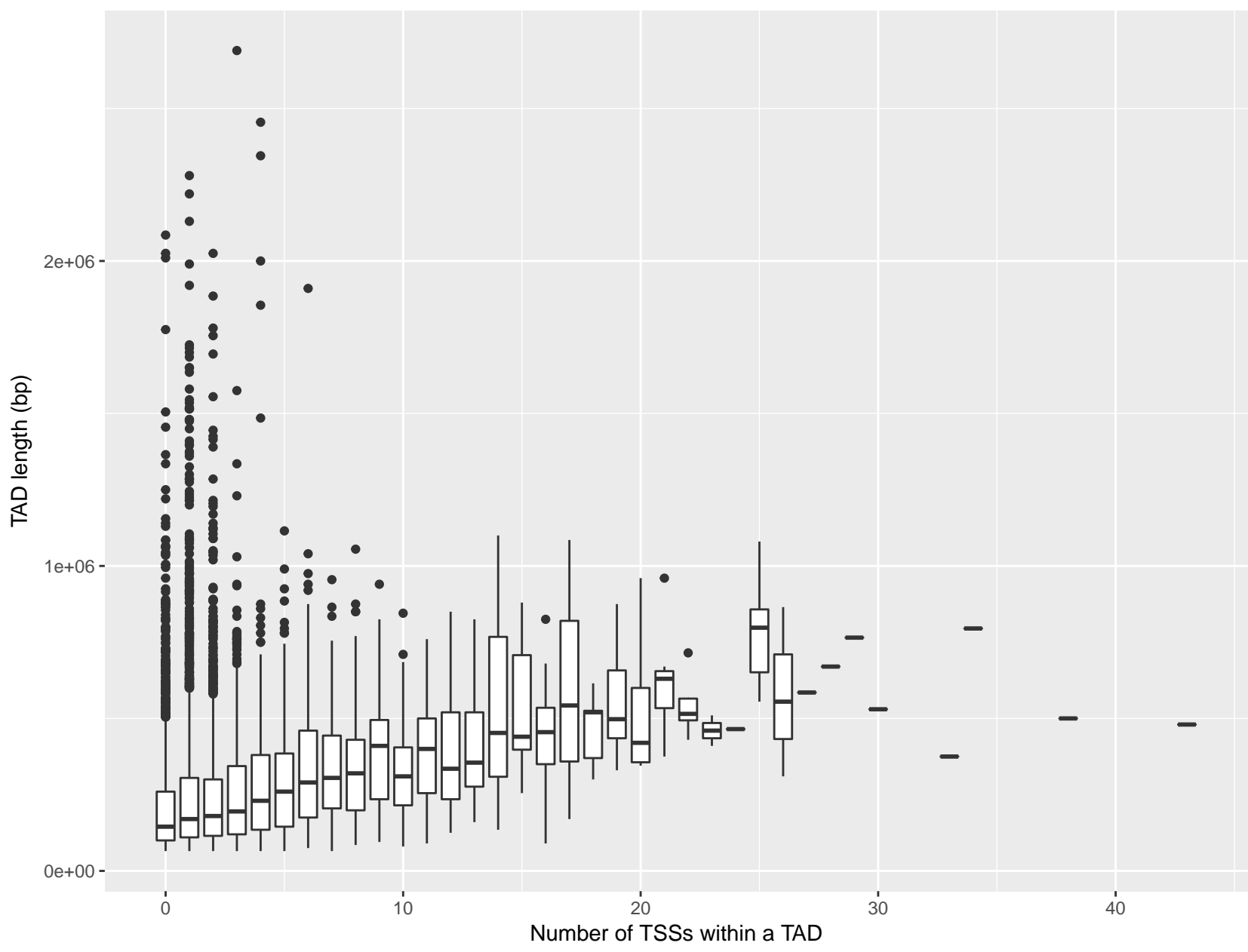

Supplement: Supplementary file 9 — Additional file 9: Figure S8. Distribution of TAD lengths depending on the number of TSSs they contain. An horizontal black line indicates the median for each TAD category. [file 13072_2019_317_MOESM9_ESM.pdf]
